# Alterations in Epoxyeicosatrienoic Acids Metabolism in Type 2 Diabetes Mellitus and Potential Association with Retinopathy Progression

**DOI:** 10.2174/0109298673474072260623040834

**Published:** 2026-07-13

**Authors:** İsmail Sarı, Esma Özmen, Kürşad Ramazan Zor, Serpil Erşan, Erkut Küçük, Durmuş Ayan, İsmail Abasıkeleş

**Affiliations:** 1 Department of Medical Biochemistry, Faculty of Medicine, Kırklareli University, Kırklareli, 39100, Türkiye;; 2 Department of Medical Biochemistry, Faculty of Medicine, Nigde Omer Halisdemir University, Niğde, 51240, Türkiye;; 3 Department of Ophthalmology, Faculty of Medicine, Nigde Omer Halisdemir University, Niğde, 51240, Türkiye;; 4 Department of Internal Medicine, Nigde Omer Halisdemir Education and Research Hospital, Niğde, Türkiye

**Keywords:** Cytochrome P450 epoxygenases, diabetic retinopathy, epoxyeicosatrienoic acid, soluble epoxide hydrolase, type 2 diabetes mellitus, metabolism

## Abstract

**Introduction:**

Epoxyeicosatrienoic acids (EETs), synthesized by cytochrome P450 epoxygenases (mainly CYP2C9, CYP2J2, and CYP2S1), are vasoprotective lipid mediators that are rapidly degraded by soluble epoxide hydrolase (sEH). This study aimed to examine changes in EET metabolism in T2DM patients with and without DR to understand its potential role in disease pathogenesis and progression.

**Methods:**

Two hundred T2DM patients (without DR), 100 with non-proliferative DR (NPDR), 100 with proliferative DR (PDR), and 200 matched healthy controls were enrolled in the study. Plasma EET levels were quantified using LC-MS/MS, and mRNA and protein levels of CYP2J2, CYP2C9, CYP2S1, and sEH were measured using qPCR and ELISA, respectively.

**Results:**

Compared to the controls, T2DM without DR and NPDR groups, patients with PDR had significantly higher EET levels (*p* < 0.05). In contrast, the T2DM without DR and NPDR groups exhibited remarkably lower EET levels than controls, although this was not significant. In both groups, CYP2J2 and CYP2S1 mRNA and/or protein levels were significantly lower, suggesting suppressed EET biosynthesis. However, CYP2C9 protein levels were higher in NPDR and PDR groups than in the control group. Furthermore, in the comparison between NPDR and PDR, CYP2J2 and CYP2S1 mRNA levels were higher in PDR, whereas CYP2C9 and EPHX2 expression did not differ significantly.

**Discussion:**

Plasma EET concentrations showed a tendency to be lower in the T2DM without DR and NPDR groups compared with healthy controls; however, these differences did not reach statistical significance. In contrast, patients with PDR exhibited significantly higher EET levels, suggesting stage-dependent alterations in EET metabolism during the progression of DR.

**Conclusion:**

Altered EET metabolism may play a dual role in DR, influencing both early microvascular impairment and late-stage angiogenic progression.

## 1 INTRODUCTION

Diabetic cardiomyopathy, nephropathy, and retinopathy are well-recognized complications of both type 1 and type 2 diabetes mellitus (DM) [1]. Diabetic retinopathy (DR) is a severe microvascular complication of DM that can lead to vision loss in advanced stages [2, 3]. DR is the leading cause of newly diagnosed blindness in the working-age population [4]. This disease is classified into two main categories: nonproliferative diabetic retinopathy (NPDR), characterized by the absence of vasoproliferation, and proliferative diabetic retinopathy (PDR), characterized by pathological neovascularization. The earliest clinical manifestations of NPDR are microaneurysms and retinal hemorrhage. Other symptoms include retinal venous loops, hard and soft exudates, and macular edema [2, 3]. In patients with PDR, neovascularization of the optic disc and/or retina, preretinal hemorrhage, vitreous hemorrhage, tractional retinal detachment, and rubeosis iridis were observed in the study. Approximately 50% of patients with untreated severe NPDR progress to PDR within one year [4, 5].

Epoxyeicosatrienoic acids (EETs) are bioactive molecules synthesized through the epoxidation of arachidonic acid by cytochrome P450 (CYP) epoxygenases, mainly belonging to the CYP2C, CYP2J, and CYP2S1 subfamilies [6, 7]. EETs exhibit vasodilatory and anti-inflammatory properties and play roles in blood pressure regulation and antioxidant defense [8-10]. Soluble epoxide hydrolase (sEH), encoded by the human EPHX2 gene, metabolizes EETs into less biologically active dihydroxyeicosatrienoic acids (DHETs), thereby modulating and limiting their biological effects. EETs are also considered endothelium-derived hyperpolarizing factors. They affect insulin release and sensitivity and exhibit proliferative and angiogenic effects [8-11]. Additionally, pancreatic islet cells have been shown to possess the capacity to produce EETs *in vivo* [12]. Moreover, sEH activity, which degrades EETs, has been reported in the small blood vessels of organs affected by diabetes-related microvascular complications [13, 14]. Collectively, these findings suggest that EETs may significantly affect glucose metabolism, type 2 diabetes mellitus (T2DM), and its associated complications. Numerous studies, mainly based on animal models, have demonstrated associations between EETs and insulin sensitivity [15], diabetes mellitus [16], and nephropathy [17]. Furthermore, EET metabolism has been implicated in cardiovascular disease, hypertension, and stroke [8, 9]. Similar to these diseases, hypertension, inflammation, and endothelial dysfunction are involved in the pathogenesis of DR. Hypertension has been suggested to contribute to DR development by inducing endothelial damage and vascular wall tension [18]. Therefore, individual variations in EET metabolism and/or levels may play a significant role in the development of DM and related complications.

To the best of our knowledge, aside from a few animal studies, no comprehensive investigation has been conducted on EET metabolism in patients with DR, and our knowledge of this topic remains limited. Therefore, we aimed to comprehensively examine the possible relationship between EET metabolism and DR. Specifically, we measured plasma EET levels in individuals with T2DM, with and without DR complications. Additionally, we quantified the expression levels of sEH, the enzyme responsible for EET degradation, and CYP2J2, CYP2C9, and CYP2S1, which are the key enzymes involved in EET biosynthesis, at both the protein and mRNA levels.

## 2 MATERIALS AND METHODS

### 2.1 Study Population

Two hundred patients diagnosed with T2DM according to the American Diabetes Association criteria [19] were recruited from the Endocrinology and Metabolism outpatient clinic at Niğde Ömer Halisdemir University Training and Research Hospital, and these patients had been followed as diabetic patients for at least 10 years. Patients who did not receive a diagnosis of diabetic retinopathy (DR) based on fundus examination at the ophthalmology clinic were classified into the “T2DM without DR” group. A total of 100 patients diagnosed with non-proliferative diabetic retinopathy (NPDR) and 100 patients diagnosed with proliferative diabetic retinopathy (PDR) were assigned to the NPDR and PDR groups, respectively. PDR and NPDR were diagnosed using fundus examination, optical coherence tomography (OCT; Cirrus 4000 HD-OCT, Zeiss Meditec), and fundus fluorescein angiography. Care was taken to ensure that the age, sex, and body mass index (BMI) distributions of the control, T2DM, NPDR, and PDR groups were matched. Participants were excluded from all three patient groups if they had any retinal disorder other than DR, if they had comorbid conditions such as diabetic nephropathy, coronary artery disease, malignancy, organ failure, polycystic ovary syndrome, ulcerative colitis, inflammatory disorders, pregnancy, renal disease, or systemic autoimmune diseases, including rheumatoid arthritis, and if they had hormonal disorders other than T2DM and/or obesity.

The control group comprised 200 healthy volunteers who were not diagnosed with DM and had no chronic disease. Pregnant women and individuals with insulin resistance or prediabetes were excluded from the control group. The authors complied with the ethical standards laid down in the 1964 Declaration of Helsinki, and this study was approved by the Erciyes University Clinical Research Ethics Committee (Decision No: 2019/102). Informed consent was obtained in writing from all individuals included in this study.

### 2.2 Collection of Blood Samples

Five mL of blood was collected from each participant into two sterile EDTA tubes. Blood samples were collected from all participants after a 12-hour fast. Approximately 1 mL of each sample was used for total RNA isolation, and the remaining blood samples were centrifuged at 3000 rpm for 10 min to separate the plasma. Plasma samples were stored at -80°C until analyses of enzyme and EET levels were performed.

### 2.3 RNA Extraction and cDNA Synthesis

Total RNA was isolated from peripheral blood samples using the QIAamp RNA Blood Mini Kit (Qiagen, Germany), according to the manufacturer's protocol. RNA was extracted immediately after sample collection to prevent the degradation of RNA. The purity of the extracted RNA was assessed using a microplate reader (Epoch, BioTek, USA) to ensure an A260/A280 ratio of ~1.8. High-quality RNA samples were used immediately for the cDNA synthesis. cDNA synthesis was performed using a RevertAid First Strand cDNA Synthesis Kit (Thermo Scientific, USA) following the manufacturer’s protocol. The synthesized cDNA and remaining RNA samples were stored at -80°C.

### 2.4 qPCR for *EPHX2, CYP2J2, CYP2S1* and *CYP2C9*

cDNAs were amplified using a Rotor-Gene 6000 real-time PCR system (Qiagen, Hilden, Germany). Reactions were performed using gene-specific, optimized, and validated primer sets targeting human *EPHX2, CYP2J2, CYP2S1, CYP2C9*, and *GAPDH* (QuantiTect Primer Assay, Qiagen) in combination with RT^2^ SYBR Green qPCR Mastermix (Qiagen, Hilden, Germany) following the manufacturer's protocols.

The thermal cycling conditions consisted of an initial denaturation and enzyme activation step at 95°C for 10 min, followed by 40 amplification cycles of denaturation at 95°C for 15 s and annealing/extension at 60°C for 60 s, during which fluorescence data were collected using the ‘Green’ detection channel. Negative controls were included in each run to monitor contamination, and GAPDH was used as an internal reference (housekeeping) gene. Relative gene expression levels were calculated using the 2^−ΔΔCt^ method, and data were analyzed using the GeneGlobe Data Analysis Center (Qiagen; https://www.qiagen.com/tr/gene-globe). The specificity of the PCR products was confirmed using melting curve analysis.

### 2.5 Quantification of Plasma sEH, CYP2J2, CYP2C9, and CYP2S1 Enzyme Levels by ELISA

The enzyme levels of human sEH, CYP2J2, CYP2C9, and CYP2S1 in plasma samples were measured using ELISA kits (catalog numbers E3882Hu, E7614Hu, E5160Hu, and E7618Hu; Bioassay Technology Laboratory, Shanghai, China) according to the manufacturer’s instructions. The intra-assay coefficient of variation (CV) was <8%, and the inter-assay CV was <10%.

### 2.6 Determination of Plasma EET Levels

The reference standards for (±)5(6)-epoxy-8Z,11Z, 14Z-eicosatrienoic acid (5,6-EET; Cayman Chemical, cat. no. 50211), (±)8(9)-epoxy-5Z,11Z,14Z-eicosatrienoic acid (8,9-EET; Cayman Chemical, cat. no. 50351), (±)11(12)-epoxy-5Z,8Z,14Z-eicosatrienoic acid (11,12-EET; Cayman Chemical, cat. no. 50511), and (±)14(15)-epoxy-5Z,8Z,11Z-eicosatrienoic acid (14,15-EET; Cayman Chemical, cat. no. 50651) were obtained from Cayman Chemical (Ann Arbor, MI, USA). The deuterated internal standard 14,15-EET-d11 was also purchased from the same supplier (Cayman Chemical cat no. 10006410). LC-MS grade methanol, water, formic acid, ammonium formate, and dichloromethane were obtained from Merck (Darmstadt, Germany).

Chromatographic separation was performed using a Kinetex C18 column (2.6 μm particle size, 50 × 3 mm internal diameter; Phenomenex, Le Pecq, France).

All chemical reagents and organic solvents were handled by trained laboratory personnel in accordance with institutional laboratory safety procedures. Appropriate personal protective equipment was used throughout the sample preparation and analysis.

Plasma EET concentrations were quantified using a liquid chromatography-tandem mass spectrometry system (LC-MS/MS, Shimadzu 8040) coupled to a Shimadzu Prominence UFLC system in the laboratory of Bome Trivitron Industry Products Foreign Trade Inc., following a validated analytical procedure previously described [20, 21].

### 2.7 Statistical Analysis

The data obtained from the study were analyzed using SPSS (Version 18). Kolmogorov-Smirnov and Shapiro-Wilk tests were performed to assess whether the data followed a normal distribution. Based on these results, Kruskal-Wallis and Mann-Whitney U tests were used to compare EET, CYP2J2, CYP2S1, sEH, and CYP2C9 protein levels between the groups. A one-way analysis of variance (ANOVA) test was conducted to compare age and BMI between the study groups. Statistical significance was set at *P* ≤ 0.05.

## 3 RESULTS

### 3.1 Demographic and Clinical Characteristics of the Study Groups

The demographic and clinical characteristics of the study groups are summarized in Table **1**. No statistically significant differences were observed in the mean age, sex distribution, and BMI between the control, T2DM without DR, NPDR, and PDR groups (*P*>0.05). Fasting blood glucose (FBG) and HbA1c levels were significantly higher in the NPDR, PDR, and T2DM without DR groups compared to the control group. There were no significant differences between the NPDR, PDR, and T2DM without DR groups.

Furthermore, smoking status was categorized as non-smokers (including never and former smokers) and smokers (including occasional and daily smokers), and there was no statistically significant difference between the groups in terms of smoking status.

Finally, the duration of type 2 diabetes mellitus differed significantly among the groups (*P* = 0.001). Patients with diabetic retinopathy had a significantly longer diabetes duration than those without retinopathy, and the longest duration was observed in the PDR group (18.9 ± 9.7 years) compared with the NPDR group (14.9 ± 7.7 years).

### 3.2 mRNA Levels of *EPHX2, CYP2J2, CYP2S1, and CYP2C9*

Melting curve analysis confirmed the specificity of the qPCR for each gene (Fig. **S1**). The relative expression levels of *EPHX2, CYP2C9, CYP2J2*, and *CYP2S1* in the study group compared to those in the control group are shown in Fig. (**1**).

*EPHX2* expression was 1.27-fold higher in the PDR group than in the control group, although this increase was not statistically significant. No significant differences in gene expression were observed among the other groups compared to the controls.

*CYP2C9* expression was significantly lower (1.67 fold) in the T2DM without DR group and 1.75 fold higher in the NPDR group than in the controls (*p*<0.05). The 1.48-fold increase in the PDR group was not significant.

*CYP2J2* expression was significantly lower (1.65 fold) in the NPDR group than in the controls, whereas the observed reductions in the T2DM without DR and PDR groups compared to the control group were not statistically significant.

Compared to the controls, *CYP2S1* expression was significantly reduced by 1.61-fold in the T2DM without DR group and 1.74-fold in the NPDR group, whereas the reduction in the PDR group was not significant.

When we analyzed gene expression in the NPDR and PDR groups compared with the T2DM without DR group, we observed that CYP2C9 expression levels were 2.74- and 2.30-fold higher in the NPDR and PDR groups, respectively (*p*<0.05). However, compared with the T2DM without DR group, no significant differences in mRNA levels of EPHX2, CYP2S1, and CYP2J1 were observed in the NPDR and PDR groups (Table **2**).

Finally, when comparing the NPDR and PDR groups, the *CYP2J2* and *CYP2S1* levels in the PDR group were significantly higher (1.58- and 1.49-fold, respectively) than those in the NPDR group. No significant differences were observed in *EPHX2* and *CYP2C9* mRNA levels between the two groups.

### 3.3 Plasma Levels of sEH, CYP2J2, CYP2C9, and CYP2S1 Proteins

Significant differences were observed in the plasma levels of sEH, CYP2J2, CYP2C9, and CYP2S1 between the groups (*p* < 0.05, Table **3**). Pairwise comparisons revealed that CYP2J2 levels were significantly lower in the T2DM without DR group than in the control group. However, no significant differences were observed in the other three enzymes between the two groups. Compared with the controls, CYP2C9 levels were significantly higher, whereas CYP2J2 and CYP2S1 levels were lower in the NPDR group. However, no significant difference was observed in sEH levels between the NPDR and control groups. Compared with the control group, the PDR group had significantly higher sEH and CYP2C9 levels and lower CYP2J2 levels. No significant difference was observed in CYP2S1 levels between the PDR and control groups.

When comparing the T2DM without DR and NPDR groups, no significant difference was found between the two groups in terms of sEH levels. However, CYP2C9 levels were significantly higher, whereas CYP2J2 and CYP2S1 levels were significantly lower in the NPDR group.

Compared with the T2DM without DR group, sEH and CYP2C9 levels were significantly higher in the PDR group, whereas CYP2J2 levels were lower. No significant difference was observed between the two groups in terms of CYP2S1 levels.

When the NPDR and PDR groups were compared, sEH and CYP2J2 levels were significantly higher in the PDR group. However, no differences were observed between the two groups in terms of CYP2C9 and CYP2S1 levels.

### 3.4 Plasma EET Levels

LC-MS/MS analysis confirmed the specific separation of all analytes within 7.5 min, with peaks for 8,9-, 11,12-, and 14,15-EET appearing between 2.8-3.2 min (Fig. **S2**). There were significant differences in plasma EET levels between the study groups (Fig. **2**).

The 8,9-EET, 11,12-EET, and 14,15-EET levels were significantly higher in the PDR group than in the control, T2DM without DR, and NPDR groups. However, no significant differences in any of the three EET isomers were observed between the control and T2DM without DR groups, the NPDR and T2DM without DR groups, and the control and NPDR groups.

When EET levels were analyzed within each group based on sex, it was observed that in the T2DM without DR group, 8,9-EET (*p* < 0.05) and 11,12-EET (*p* < 0.05) levels were significantly higher in males than in females. Specifically, the median 8,9-EET levels were 77.2 (0.1-789.2) in males and 27.4 (0.0-809.9) in females, whereas the median 11,12-EET levels were 171.8 (1.5-1796.4) and 32.5 (0.0-1904.2), respectively. No statistically significant sex-related differences were observed among the other groups.

## 4 DISCUSSION

Studies investigating EET metabolism in patients with diabetes and/or associated complications in humans are limited, and some findings remain contradictory. In this study, we comprehensively examined EET metabolism at both the molecular and biochemical levels in patients with T2DM and DR. Our findings suggest that EET metabolism undergoes remarkable alterations in patients with T2DM, NPDR, and especially PDR.

We found that CYP2C9 and CYP2S1 gene expression, which are involved in EET biosynthesis, were significantly lower in the T2DM without DR group (*p* < 0.05). Similarly, ELISA results showed a notable but non-significant decrease in CYP2C9 protein levels. Consistent with these data, two separate studies in rodent models of insulin resistance reported a reduction in hepatic and vascular CYP2C expression and activity [22, 23]. Furthermore, we found that CYP2J2 protein levels were significantly lower in the T2DM without DR group. However, the 1.31-fold reduction in CYP2J2 gene expression was not statistically significant. Our results from protein and gene expression analyses suggest a trend toward reduced EET synthesis in T2DM patients without DR. Consistent with this, we observed a marked decrease in EET levels. However, because the decrease in plasma EET levels did not reach statistical significance, the data only support a possible alteration in EET bioavailability.

Similarly, Duflot *et al.* reported that EET and DHET regioisomers, and total plasma EET + DHET levels, were slightly lower in T2DM patients than in healthy controls, but the differences between groups were not statistically significant [16]. In different studies conducted with diabetic animal models, it was found that diabetic animals have lower EET levels [24], and inhibition or deletion of sEH, which leads to increased EET levels, improved insulin secretion and sensitivity, and reduced glucose levels [25, 26]. sEH inhibition has been shown to improve endothelial function and metabolic homeostasis in various studies [24, 27]. Furthermore, plasma levels of 8,9-EET, 11,12-EET, and 14,15-EET have been found to correlate with insulin sensitivity in humans. It has been suggested that EETs enhance insulin sensitivity and vascular reactivity and lower blood pressure [15]. Considering these studies and our findings, altered EET signaling may be relevant to the pathophysiology of T2DM rather than indicating a definitive decrease in EET levels.

In our study, CYP2C9 expression was significantly higher in patients with NPDR than in healthy controls, whereas CYP2J2 and CYP2S1 expression levels were lower at both the mRNA and protein levels. There was no statistically significant difference between the NPDR group and controls in terms of sEH enzyme levels. Although EET levels were markedly lower in the NPDR group, this difference was not statistically significant. Some studies have demonstrated that the use of EET analogs or increased EET synthesis reduces reactive oxygen species and proinflammatory cytokine levels while improving insulin sensitivity [28, 29]. Furthermore, data from two different studies conducted in mouse models suggest that EET metabolism plays a role in NPDR pathogenesis, supporting our hypothesis. These studies demonstrated that treatment with a specific sEH inhibitor prevented pericyte loss and vascular permeability, which are characteristic features of NPDR [13, 30]. These findings support the hypothesis that preserving EET bioactivity is protective against early microvascular damage in DR. On the other hand, it has been reported that EETs induce VEGF expression *via* a potential positive feedback mechanism, which further enhances endothelial cell proliferation and angiogenesis [31]. This phenomenon is thought to be related to the ability of EETs to activate hypoxia-inducible factor alpha (HIF-1α) [32]. In addition, it was shown that VEGF activates the CYP2C promoter, increasing its expression and activity, which causes an increase in intracellular EET levels in endothelial cells [33]. These findings raise the question of whether increasing EET levels as a therapeutic approach for diabetic retinopathy might contribute to the progression of advanced-stage DR (PDR), which is typically characterized by retinal hypoxia and increased VEGF levels. Therefore, modulation of EET signaling may require stage-specific evaluation of DR in future studies.

In our study, CYP2C9 and sEH protein levels were significantly higher in the PDR group than in healthy controls. The mRNA levels of these genes increased by 1.48-fold and 1.27-fold, respectively, but these changes were not statistically significant. No significant differences were observed in CYP2S1 mRNA and protein levels between the two groups. Furthermore, the levels of all three EET isomers were significantly higher in the PDR group than in the control, NPDR, and T2DM without DR groups. However, when comparing the NPDR and PDR groups, CYP2J2 and CYP2S1 levels in the PDR group were significantly higher (1.58- and 1.49-fold, respectively) than in the NPDR group. No significant differences were observed between the two groups in terms of EPHX2 and CYP2C9 mRNA levels.

According to our results, circulating EET levels and the expression of enzymes involved in EET biosynthesis and degradation do not appear to be completely consistent. However, EET levels are regulated by a complex network that extends beyond CYP-mediated biosynthesis. Circulating EET concentrations may be influenced by substrate availability, cellular release from vascular and inflammatory cells, alterations in tissue-specific production, changes in metabolic degradation, and compensatory responses to chronic ischemic and inflammatory stress. Consequently, circulating metabolite levels may not directly correlate with the gene or protein expression levels of individual biosynthetic enzymes. Previous experimental studies have demonstrated that EETs possess potent angiogenic and vasoactive properties and may increase in pathological environments characterized by ischemia, hypoxia, inflammation, and endothelial dysfunction, which are highly relevant to PDR [11, 34, 35]. Therefore, the elevated EET levels observed in the PDR group may represent a compensatory or adaptive response to progressive retinal vascular injury rather than a direct reflection of increased CYP epoxygenase expression.

Collectively, the present data show non-significantly lower EET levels in T2DM without DR and NPDR and significantly higher EET levels in PDR. PDR is characterized by abnormal blood vessel growth and angiogenesis in the retina, whereas NPDR is characterized by vascular damage without significant neovascularization [36]. Given that EETs possess angiogenic properties, it is plausible that their high levels in patients with PDR may contribute to disease progression by promoting angiogenesis. Consistent with this hypothesis, CYP2C enzymes have been reported to contribute to the formation of proangiogenic metabolites from ω-6 and ω-3 long-chain polyunsaturated fatty acids. Furthermore, it has been observed that CYP2C8 and CYP2C9 levels are significantly increased in human retinal astrocyte cells under hypoxia, that CYP-derived 11,12-EET supports the proliferation and tube formation of retinal microvascular endothelial cells, and that it stimulates angiogenesis in the retina under hypoxia. Moreover, when astrocytes were treated with a CYP inhibitor, hypoxia-induced VEGF production decreased, and VEGF-induced proliferation of microvascular endothelial cells was inhibited. Therefore, it has been proposed that targeting CYP enzymes might serve as a therapeutic approach against retinal neovascularization [11]. In another study investigating pro-angiogenic mediators derived from arachidonic acid in the vitreous humor of patients with PDR, the levels of 11,12-EET, total EETs, and other eicosanoids were significantly higher in patients with PDR than in controls. The researchers in this study showed that 8,9- and 11,12-EET levels had an increasing trend, but the difference was not statistically significant. Even though EETs stand out for their cytoprotective, anti-inflammatory, and neuroprotective properties, it has been determined that they can exhibit pro-angiogenic effects in the retina through hypoxic stimulation of astrocytes [37]. Similarly, it has been reported that 14,15-EET exerts pro-angiogenic effects on pathological retinal and choroidal neovascularization [38, 39], and CYP2C8 inhibition has been found to prevent this neovascularization [38].

Taken together, these data suggest that EETs have stage-dependent associations with DR in the present study. However, in the early stages of the disease (T2DM without DR and NPDR), our data only showed non-significant downward trends in plasma EET levels; therefore, any inference regarding reduced EET bioavailability or downstream microvascular effects remains speculative.

## 5 STUDY LIMITATIONS

This study had some limitations. First, its cross-sectional design prevents any causal inference regarding the relationship between EET metabolism and DR progression. Second, EPHX2, CYP2J2, CYP2C9, and CYP2S1 mRNA and protein levels were measured in peripheral blood, and ELISA-based measurements of these largely intracellular enzymes in plasma may be derived from circulating cells and extracellular release from other tissues rather than from direct retinal expression. CYP enzymes are predominantly intracellular and membrane-associated enzymes; therefore, their detection in plasma should not be interpreted as direct evidence of retinal enzymatic activity. Rather, they may represent enzymes released into the circulation from cell turnover and/or from cells undergoing necrosis due to increased inflammation, oxidative stress, and similar events, which are particularly closely related to DR pathogenesis. Third, although blood pressure and lipid profiles are clinically important variables in DR, these parameters were not systematically collected as primary study variables in this preliminary study, which focused on the metabolism of EET. Therefore, these variables were not incorporated into the current analysis because they were not systematically collected across the entire study population at the time of study design.

Furthermore, although major confounders were minimized by matching for age, sex, and BMI and by applying exclusion criteria, residual confounding by medication use could not be excluded from this study. Medication use (*e.g.*, metformin, insulin, and statins) can influence endothelial function and EET metabolism. Although patients in our study groups received standard antidiabetic, antihypertensive, and lipid-lowering therapies, detailed medication histories were not uniformly available for all participants and were therefore not included in the statistical analysis. Future longitudinal studies, including ocular samples, detailed blood pressure and lipid profiles, and pharmacological data, are needed to clarify the mechanism and exact effect of EET metabolism on DR progression and to validate our results.

## CONCLUSION

In conclusion, our findings suggest that plasma EET levels are not significantly lower in T2DM without DR and NPDR and significantly higher in PDR. Elevated EET levels in patients with PDR may promote disease progression by enhancing angiogenesis. Although CYP2J2, CYP2C9, CYP2S1, and sEH are predominantly expressed in tissues, mainly in the liver, low but detectable levels of their mRNA and proteins can be measured in peripheral blood. Therefore, their expression levels in blood do not necessarily correlate with tissue levels. However, in this study, we measured the expression of these enzymes in peripheral blood because it is a non-invasive approach. Therefore, our expression results do not directly reflect gene expression in the retina but may be interpreted as systemic correlates of EET biosynthesis and degradation.

Finally, our study and previous research suggest that EET metabolism may be a potential therapeutic target for DR treatment. However, considering the proangiogenic effects of EETs and their role in increasing VEGF expression, it is important to consider the possibility that strategies aimed at increasing EET levels may exacerbate later stages of DR, which are characterized by retinal hypoxia, elevated VEGF levels, and increased angiogenesis. Further large-scale studies are required to validate these hypotheses and explore their potential therapeutic applications.

## AUTHORS’ CONTRIBUTIONS

The authors confirm thier contribution to the paper as follows: study conception and design: İS, EÖ, and KRZ; data collection: EÖ, KRZ, EK, İA, DA; analysis and interpretation of results: İS, EÖ, SE, KRZ, DA; draft manuscript: İS, SE, KRZ. All authors reviewed the results and approved the final version of the manuscript.

## Figures and Tables

**Fig. (1):**
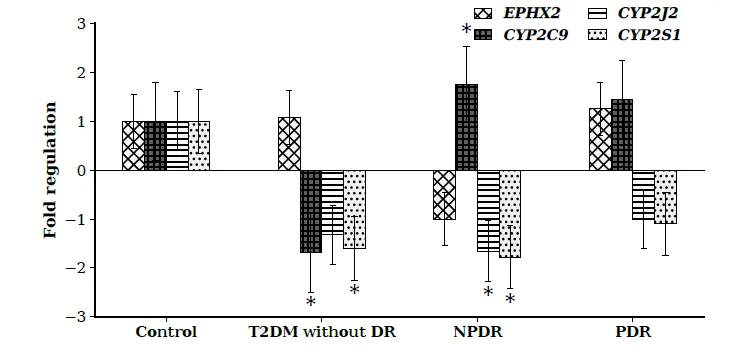
Comparison of EPHX2, CYP2J2, CYP2S1, and CYP2C9 gene expression levels in T2DM without DR, NPDR, and PDR groups with those in the control group. *p<0.05. Fold-Regulation (FR) provides a biologically meaningful interpretation of fold-change results. When the fold-change value is greater than one, it signifies upregulation, and the FR is equivalent to the fold-change. Conversely, when the fold-change value is less than one, it indicates downregulation, and FR is expressed as the negative inverse of the fold-change.

**Fig. (2):**
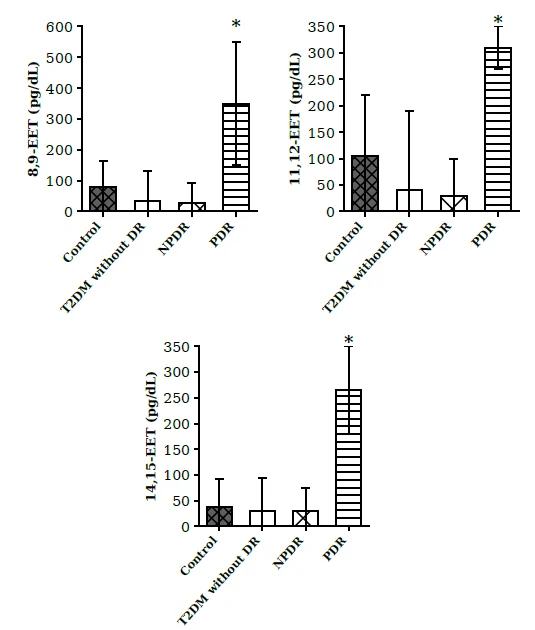
Bar graph indicating the 8,9-EET (**A**), 11,12-EET (**B**), and 14,15-EET (**C**) levels in plasma samples of the study groups. Error bars represent the 99% confidence intervals. **Abbreviations:** DR, diabetic retinopathy; EET, Epoxyeicosatrienoic acid; NPDR, nonproliferative DR; PDR, proliferative DR; T2DM, type 2 diabetes mellitus.

**Table 1:** Demographic characteristics, fasting blood sugar, and HbA1c levels of the study groups.

-	**Controls** **N=200**	**T2DM without DR** **N=200**	**NPDR** **N=100**	**PDR** **N=100**	***p* value**
Age (years) ( 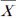 ± S)	58.4±9.6	59.6±8.4	61.1±7.7	60.9±8.4	0.110
Gender (Female/male)	114/86	135/65	56/44	54/46	0.060
BMI (Kg/m^2^, 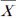 ± S)	29.4±5.7	31.1±4.0	29.5± 4.4	30.2±3.8	0.070
HbA1c (%)	5. 2±0.32	10.5±2.5	9.0±2.0	9.4±0.4	0.001*
FBG (mg/dL)	87.2±8.7	174.2±65.8	184.3±67.7	206.8±74.3	0.001*

Note: *p<0.05: Statistically significant. **Abbreviations: **FBG, fasting blood glucose; NPDR, non-proliferative diabetic retinopathy; PDR, proliferative diabetic retinopathy; T2DM, type 2 diabetes mellitus; S, standard deviation; mean.

**Table 2:** Comparison of *EPHX2, CYP2J2, CYP2S1*, and *CYP2C9* gene expression levels in the NPDR and PDR groups with those in T2DM without DR.

**Reference Group**	**Gene**	**NPDR (FR)**	***p*-value**	**PDR (FR)**	***p*-value**
T2DM without DR	*EPHX2*	-1,07	0.367	1,19	0.296
T2DM without DR	*CYP2C9*	2.74*	0.003*	2,30*	0.007*
T2DM without DR	*CYP2J2*	-1,23	0.047*	1,33	0.216
T2DM without DR	*CYP2S1*	-1,11	0.342	1,41	0.098

Note: *p<0.05: Statistically significant. **Abbreviations: ** NPDR, non-proliferative diabetic retinopathy; PDR, proliferative diabetic retinopathy; T2DM, type 2 diabetes mellitus; FR, Fold-Regulation (provides a biologically meaningful interpretation of fold-change results).

**Table 3:** Statistical comparison of plasma sEH, CYP2J2, CYP2C9 and CYP2S1 levels among study groups.

	**sEH (ng/L)**	**CYP2J2 (ng/L)**	**CYP2C9(ng/mL)**	**CYP2S1(ng/mL)**
**Controls,** Median (Min-Max)	963.9 (596.7-5109.6)	6.9 (4.5-25.3)	462.9 (256.6-2367.9)	4.6 (3.7-76.1)
**T2DM without DR,** Median (Min-Max)	1276.7 (0.0-4995.3)	6.4 (0.0-25.0)	505.6 (0.0-2347.9)	4.8 (2.0-68.9)
**NPDR,** Median (Min-Max)	1272.4 (0.0-5038.1)	1.2 (0.0-11.2)	691.9 (104.9-2393.6)	4.0 (2.0-47.1)
**PDR,** Median (Min-Max)	1526.7 (1216.7-2872.4)	1.6 (1.1-4.1)	688.9 (317.6-1291.2)	4.4 (3.5-7.1)
***p* value (Overall)**	0.0060*	0.0001*	0.0001*	0.0250*
**Controls *vs* T2DM without DR**	*p*=0.301	*p*=0.044*	*p*=0.841	*p*=0.762
**Controls *vs* NPDR**	*p*=0.079	*p*=0.0001*	*p*=0.006*	*p*=0.020*
**Controls *vs* PDR**	*p* = 0.002*	*p*=0.0001*	*p* = 0.003*	*p*=0.093
**T2DM without DR *vs* NPDR**	*p*=0.187	*p*=0.0001*	*p=*0.0001*	*p*=0.017*
**T2DM without DR *vs* PDR**	*p*=0.005*	*p*=0.0001*	*p*=0.0001*	*p*=0.104
**NPDR *vs* PDR**	*p*=0.044*	*p*=0.004*	*p*=0.978	*p*=0.388

**Note: ** *p<0.05: Statistically significant. **Abbreviations: ** CYP, Cytochrome P450; NPDR, non-proliferative diabetic retinopathy; PDR, proliferative diabetic retinopathy; sEH, soluble epoxide hydrolase; T2DM, type 2 diabetes mellitus.

## Data Availability

All the data and supporting material are available within the article.

## LIST OF ABBREVIATIONS

BMI: Body Mass Index
cDNA: Complementary DNA
CV: Coefficient of Variation
CYP: Cytochrome P450
DHETs: Dihydroxyeicosatrienoic Acids
DM: Diabetes Mellitus
DR: Diabetic Retinopathy
EETs: Epoxyeicosatrienoic Acids
LC-MS/MS: Liquid Chromatography Coupled with Tandem Mass Spectrometry
MeOH: Methanol
MRM: Multiple Reaction Monitoring
NPDR: Nonproliferative Diabetic Retinopathy
PDR: Proliferative Diabetic Retinopathy
sEH: Soluble Epoxide Hydrolase

## STANDARDS OF REPORTING

STROBE guidelines were followed.
