# Supplementary material for: Alterations in Epoxyeicosatrienoic Acids Metabolism in Type 2 Diabetes Mellitus and Potential Association with Retinopathy Progression
Source: Curr Med Chem. 2026 Jul 13;33(24):5015–26. doi: 10.2174/0109298673474072260623040834 (PMC13688410; doi:10.2174/0109298673474072260623040834)
Supplement: Supplementary file 1 [file CMC-33-24-5015_SD1.pdf]

Supplementary Material

Alterations in Epoxyeicosatrienoic Acids Metabolism in Type 2 Diabetes Mellitus and Potential Association with Retinopathy Progression

İsmail Sarı[1,*], Esma Özmen[2], Kürşad Ramazan Zor[3], Serpil Erşan[2], Erkut Küçük[3], Durmuş Ayan[2] and İsmail Abasıkeleş[4]

[1]Department of Medical Biochemistry, Faculty of Medicine, Kırklareli University, Kırklareli, 39100, Türkiye; [2]Department of Medical Biochemistry, Faculty of Medicine, Nigde Omer Halisdemir University, Niğde, 51240, Türkiye; [3]Department of Ophthalmology, Faculty of Medicine, Nigde Omer Halisdemir University, Niğde, 51240, Türkiye; [4]Department of Internal Medicine, Nigde Omer Halisdemir Education and Research Hospital, Niğde, Türkiye

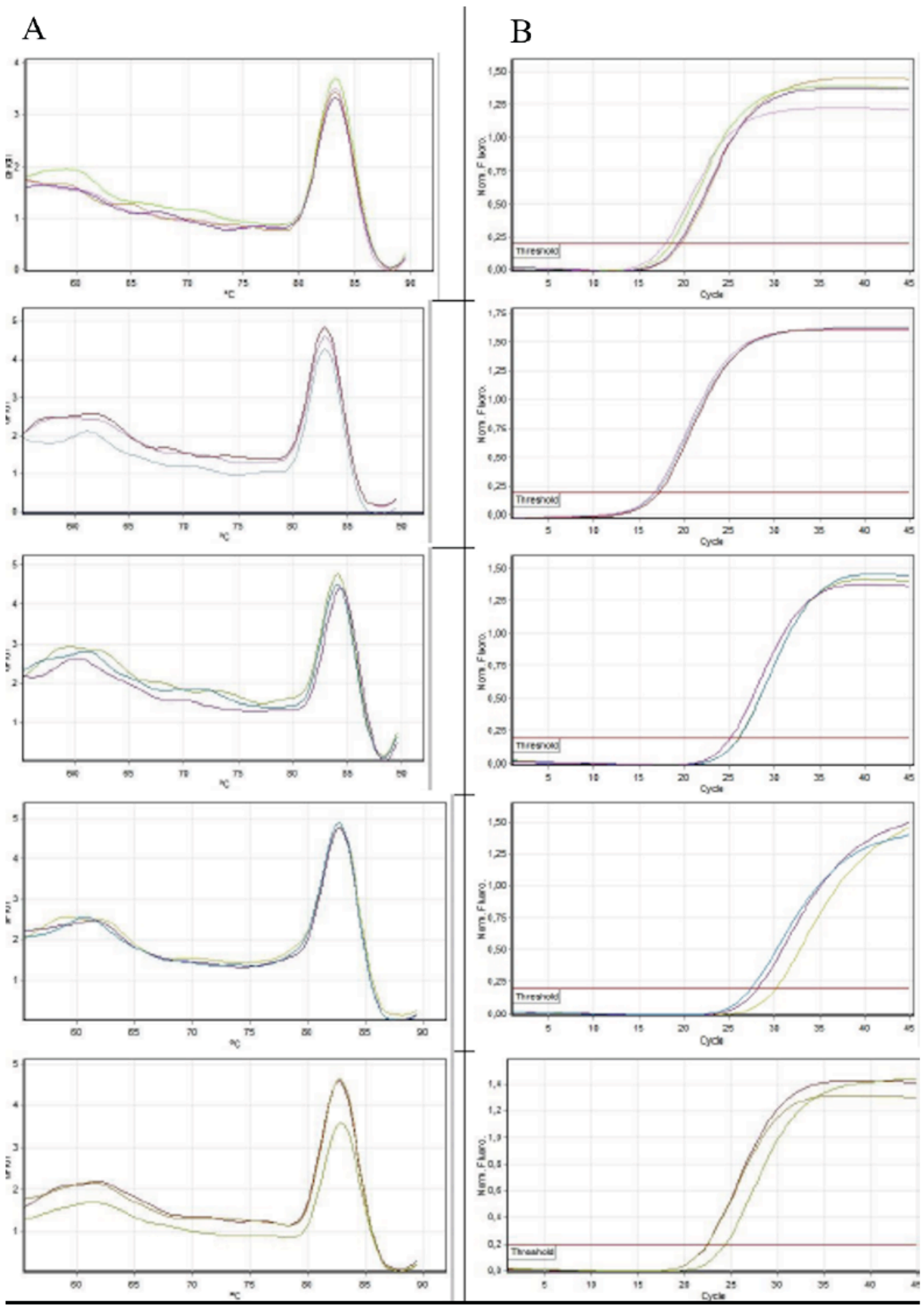

Fig. (S1).

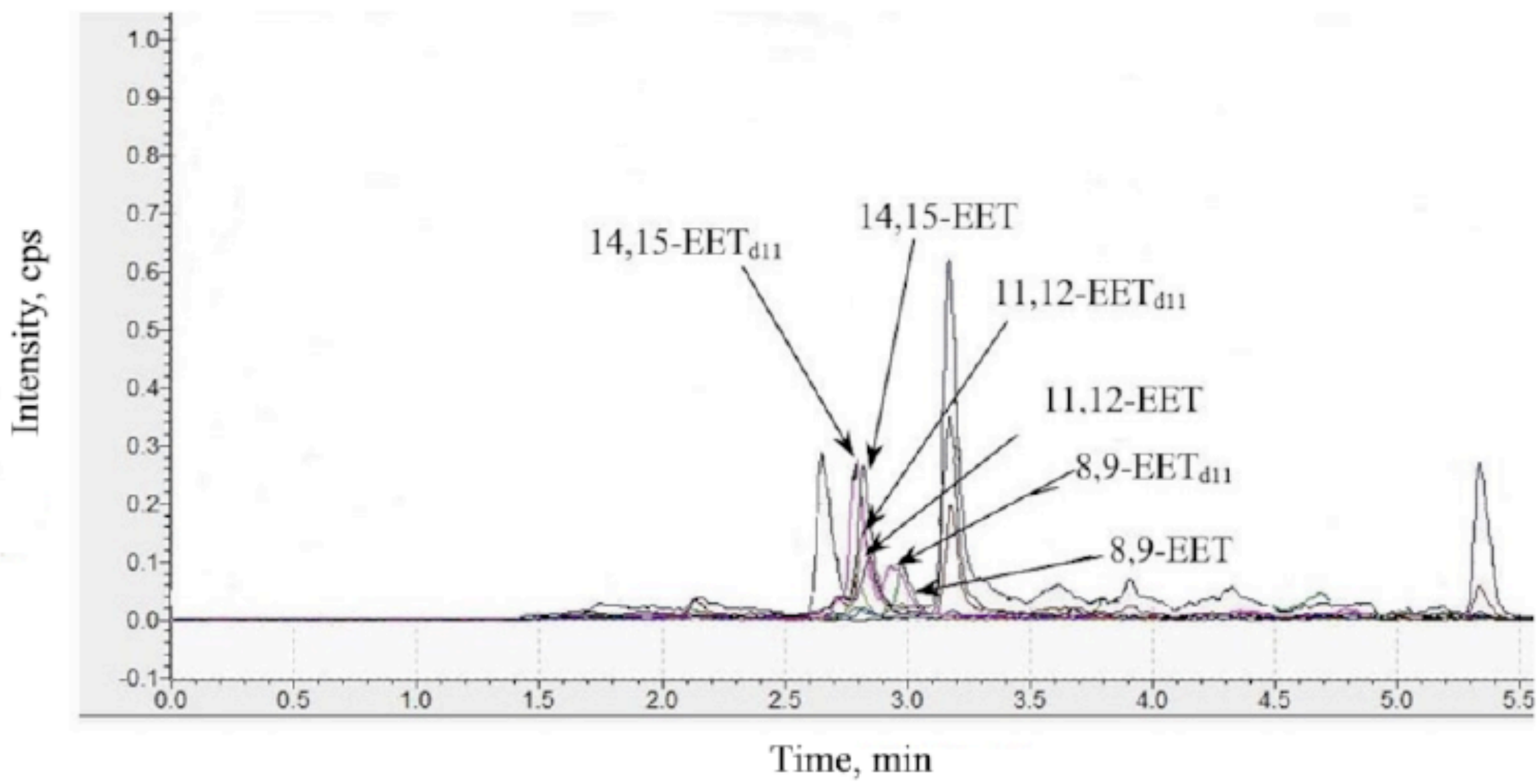
Intensity, cps
Time, min
14,15-EET$_{d11}$
14,15-EET
11,12-EET$_{d11}$
11,12-EET
8,9-EET$_{d11}$
8,9-EET
1.0
0.9
0.8
0.7
0.6
0.5
0.4
0.3
0.2
0.1
0.0
-0.1
0.0
0.5
1.0
1.5
2.0
2.5
3.0
3.5
4.0
4.5
5.0
5.5

Fig. (S2).
